# The impact of osteoarthritis and geriatric depression scale on mini-mental state examination trajectories over seven years

**DOI:** 10.1007/s10433-025-00900-x

**Published:** 2025-12-16

**Authors:** Chiara Ceolin, Marianna Noale, Sara Bindoli, Roberta Ramonda, Sabrina Pigozzo, Chiara Curreri, Adele Ravelli, Maria Devita, Giuseppe Sergi, Marina De Rui

**Affiliations:** 1 Division of Geriatrics, DIDAS Medicine Department, University – Hospital of Padua, Padua, Italy; 2 Department of Medicine, DIMED, University of Padua, Padua, Italy; 3 Department of Neurobiology, Care Sciences and Society, Aging Research Center, Karolinska Institutet and Stockholm University, Stockholm, Sweden; 4 National Research Council, Neuroscience Institute, Padua, Italy; 5 Rheumatology Unit, University – Hospital of Padua, Padua, Italy; 6 Department of General Psychology (DPG), University of Padua, Padua, Italy

**Keywords:** Osteoarthritis, Older adults, Geriatric depression scale, Cognitive decline, MMSE

## Abstract

**Abstract:**

Cognitive decline is influenced by factors such as inflammation, reduced physical activity, chronic pain, and depression. Osteoarthritis (OA), the most prevalent form of arthritis, may contribute to cognitive impairment through these mechanisms. The objectives of this study are: (1) To assess cognitive trajectories in older adults (≥ 65 years) over a 7-year period; (2) to explore the relationship between OA and cognitive decline; and (3) to investigate the potential mediating effect of depressive symptoms. Using the longitudinal dataset of Progetto Veneto Anziani (Pro.V.A), data on inflammation, and cognitive status (Mini-Mental State Examination-MMSE, Geriatric Depression Scale-GDS) were collected. OA was diagnosed based on clinical evaluations and medical records. Active follow-ups were carried out after 4.4 and 7 years from baseline. Group-based trajectory modeling identified cognitive trajectories, and multivariable logistic regression assessed factors associated with these trajectories. Structural equation modeling explored whether depressive symptoms mediated the OA-cognitive trajectories relationship. The sample included 2945 older adults (63.3% having OA). Participants with OA were older, more likely to be female, and had higher GDS and lower MMSE scores at baseline. Over 7 years, three cognitive trajectories were identified: severe cognitive decline (n = 261, 8.9%), moderate decline (n = 865, 29.3%), and stability (n = 1819, 61.8%). OA was more prevalent in participants with greater cognitive decline. Logistic regression showed that OA was significantly associated with moderate cognitive decline trajectory (OR = 1.31, 95% CI: 1.03–1.71, *p* = 0.039). OA influenced cognitive decline both directly and indirectly through depression, with depression mediating 30% of the total effect. OA seem to be associated with cognitive decline trajectory directly and indirectly through depression, highlighting the need to address mental health in OA management.

**Key points:**

- Osteoarthritis is linked to moderate cognitive decline in older adults, with depression acting as a partial mediator.
- About 30% of OA’s total effect on cognition is explained by depressive symptoms.
- Early intervention targeting both physical and psychological health may help prevent cognitive deterioration in this population.

**Supplementary Information:**

The online version contains supplementary material available at 10.1007/s10433-025-00900-x.

## Introduction

Cognitive decline is common among older adults and arises from multiple factors, including chronic inflammation, sedentary behavior, and reduced physical activity (*Risk Factors for Dementia—2024 Update The 2024 Update to the Standing Lancet Commission on Dementia Prevention, Intervention, and Care Adds Two New Risk Factors (High LDL Cholesterol and Vision Loss)*, 2024). Osteoarthritis (OA), the most prevalent degenerative joint disease, has recently emerged as a potential contributor to cognitive impairment. Evidence suggests that individuals with OA may face a higher risk of cognitive decline and dementia, possibly due to physical inactivity and mobility limitations (Falck et al. 2018; S.-W. Huang et al. 2015). Beyond functional impairment, OA involves chronic low-grade inflammation, which may contribute directly to neurodegenerative processes. Elevated inflammatory markers have been linked to poorer cognitive outcomes even in midlife (Gimeno et al. 2008), and preclinical studies indicate a mechanistic connection between OA-related inflammation and Alzheimer’s disease pathology (Kyrkanides et al. 2011). OA and cognitive decline also share several risk factors—such as age, sex, and lifestyle behaviors—supporting the hypothesis of a common pathophysiological background (Baker et al. 2017). Clinical findings are consistent with this association: Bartolini et al. reported that 38% to 70% of individuals with arthritis exhibit cognitive deficits, depending on the domain assessed (Bartolini et al. 2002), while meta-analyses confirm an increased dementia risk among OA patients (Guo et al. 2022). However, the literature is constrained by design limitations—predominantly cross-sectional analyses and cohorts with short follow-up—and by age-heterogeneous samples that are not focused on older adults. These issues reduce the robustness of prior findings and highlight the need for longitudinal studies in older populations.

Chronic pain, a core symptom of OA, plays a key role in this relationship. It has been associated with impairments in attention, executive function, and working memory (Abeare et al. 2010; Dick & Rashiq 2007). Pain may directly burden cognitive resources and indirectly affect cognition by promoting depressive symptoms (Brown et al. 2002). Depression is especially relevant in OA populations, affecting around 20% of patients (Callhoff et al. 2024), though often underdiagnosed due to overlapping physical symptoms (Wang & Ni 2022). As an established independent risk factor for cognitive decline (Devita et al. 2022), depression may act both as a consequence of pain and as a mediator of its cognitive effects.

Taken together, these findings suggest a complex interplay between OA, pain, depression, and cognitive dysfunction. Understanding these interconnections is crucial for identifying vulnerable individuals and developing integrated interventions to preserve cognitive health and improve quality of life in OA patients. Despite growing evidence, most existing studies are cross-sectional, limiting insights into how cognitive function evolves over time in this population. No research to date has examined longitudinal trajectories of Mini-Mental State Examination (MMSE) scores in relation to both OA and depressive symptoms.

Therefore, the present study aims to: 1) assess cognitive trajectories over a 7-year follow-up in older adults; 2) investigate the association between OA and these trajectories; and 3) explore the potential mediating role of depression, as measured by the Geriatric Depression Scale (GDS). We hypothesized that, in older adults, baseline OA would be independently associated with a higher probability of membership in cognitive-decline trajectories during the follow-up. We further hypothesized that depressive symptoms would contribute to the OA–cognition association.

## Materials and methods

### Study population

This study utilizes data from the Progetto Veneto Anziani (Pro.V.A.), a multicentric prospective cohort study that included individuals aged 65 years and older residing in the provinces of Padova and Rovigo in northern Italy. Participants were randomly selected using a multistep enrollment process stratified by age and sex (for further details, see (Corti et al. 2002)). The final sample included 3099 individuals (1245 M, 1854 F). Baseline assessments were conducted between 1995 and 1997 by trained nurses and physicians. Active follow-ups were carried out after a mean of 4.4 and 7 years from baseline, while passive follow-up on hospitalizations and mortality was conducted until 2018 through linkage with regional registers. The study protocol was approved by the Ethics committees of the University of Padova, the Local Health Units no.15 and 18 of the Veneto Region, and the province of Padova. All participants provided informed consent. The study complies with the principles outlined in the Declaration of Helsinki. This research was conducted as part of the Next Generation EU-funded project “Age-It—Ageing well in an ageing society” (PE0000015), under Italy’s National Recovery and Resilience Plan (NRRP)—PE8, Mission 4, Component 2, Intervention 1.3.

For the current study, we excluded 154 individuals due to incomplete baseline data on the MMSE or OA, resulting in a final analytical sample of 2945 older adults.

### Data collection

We specifically considered the following data, that were collected at baseline:

- *Sociodemographic information* (age, sex, living arrangement [living with others, living alone, living in nursing home]), educational level (categorized as ≤ 5 vs > 5 years), smoking habits (classified as never, former, and current smokers), and alcohol consumption (defined as no/occasional, light to moderate drinking with < 7 units of alcohol [UA]/week for women and < 14 UA/week for men, and heavy drinking with ≥ 7 UA/week for women and ≥ 14 UA/week for men). Regular physical activity was defined as ≥ 4 h/week in the previous month of at least moderate physical activity (brisk walking, cycling, gardening, dancing, or physical exercising). Anthropometric measures, including body weight and height, were obtained, and the body mass index (BMI) was calculated as the ratio of weight to height squared. Trained physicians ascertained the presence of comorbidities. The total number of chronic conditions for each participant was used as an indicator of multimorbidity. Multimorbidity was defined as the presence of two or more chronic diseases (Alvarez-Galvez & Vegas-Lozano 2022).
- *Functional status* was measured through the Activities of Daily Living (ADL) scale, assessing self-sufficiency in bathing, dressing, transferring, toileting, continence, and feeding (Katz 1983).
- *Inflammatory Status* Biochemical markers were obtained from blood samples collected after overnight fasting and analyzed at the central laboratory of the city hospital following standard quality-controlled procedures. Inflammatory status in our study was assessed by measuring the serum levels of fibrinogen, erythrocyte sedimentation rate, and white blood cells. In the whole sample, the inflammatory status was classified as "low" if at least one of the three biochemical measurements was below the median value (339.00 mg/dL for fibrinogen, 15 mm/h for erythrocyte sedimentation rate and 5.80 × 109/L for white blood cells).

#### Outcome

The Mini-Mental State Examination (MMSE) (Folstein et al. 1975; Magni et al. 1996) was assessed both at baseline and at the two follow-up periods. The MMSE consists of 30 questions that evaluate seven cognitive domains: orientation to time and place, word recall, attention and calculation, recall, language, and constructional praxis. Scores range from 0 to 30, with a score below 24 indicating the presence of cognitive deficits. The raw MMSE scores were used, without adjustment for age and education levels. Each participant contributed up to 3 MMSE measurements during the study period (baseline, data available for n = 2945 participants; first follow-up, data available for n = 2143; second follow-up, data available for n = 623).

#### Exposure

The presence of osteoarthritis was recorded at baseline and at the two follow-ups. As previously reported (Veronese et al. 2016), OA involving the hand, hip, and knee was assessed on the grounds of participants’ medical history and records, previous X-ray reports, the use of specific analgesics, and clinical examinations that included the following: for the hand, any presence of Heberden nodes, stiffness, and pain on passive moment; for the knee, deformity, pain on passive movement, limited passive mobility, and crepitus; and for the hip, pain on passive movement, rotation and palpation, and limited external rotation. Any diagnosis of OA was confirmed by a rheumatologist using a standardized algorithm for investigating signs and symptoms of OA, X-ray reports, previous hospitalizations for OA, and prostheses for OA-related complications.

Potential mediators.

Depressive symptoms were measured using the 30-item GDS at baseline. The GDS score ranges from 0 (no depressive symptoms) to 30 (depressive symptoms), with a cutoff of 11 indicating clinically significant depressive symptoms (Yesavage et al., n.d.).

### Statistical analysis

Baseline characteristics of the study participants are presented as mean (standard deviation [SD]), median (interquartile range [IQR]), and count (frequency) for quantitative and qualitative variables, respectively. Comparisons of these characteristics between participants with and without OA at baseline were conducted using generalized linear models (Levene’s test was used to assess homoschedasticity, and Welch’s ANOVA was used in the presence of heteroschedasticity), the Wilcoxon rank-sum test, and either the Fisher exact test or Chi-squared test, as appropriate.

Group-based trajectory modeling was performed using SAS Proc Traj to identify subgroups of participants with similar MMSE trajectories (Jones et al. 2001). This method accommodates participants with incomplete follow-up data under the assumption that data are missing at random, thus enabling the use of all available information without need for imputation (S. Huang et al. 2024; Nagin & Odgers 2010). A censored normal distribution, recommended for continuous outcomes with natural upper and lower bounds such as MMSE scores, was applied. The optimal model was selected based on the following criteria:

- Model fit statistics, specifically the Bayesian Information Criterion (BIC); the magnitude of the BIC difference was used to choose between more complex and simpler models (with a difference of 2ΔBIC > 10 indicating preference for simpler models) (Jones et al. 2001);
- The significance of polynomial terms, starting with a quadratic trajectory specification and removing nonsignificant polynomial terms;
- A minimum group membership probability of 5%;
- An average posterior probability greater than 0.7 within each group.

In addition, relative entropy was calculated from posterior probabilities of group membership to assess class separation.

Final models included age and sex, as potential risk factors influencing the probability of group membership. Fisher exact test, Chi-squared test, generalized linear models or Kruskal–Wallis tests, were used to assess the unadjusted differences among the identified trajectory groups. Multivariable logistic regression models were used to assess variables associated with the cognitive trajectory groups; variables associated with MMSE trajectories were first analyzed in univariable models, and those showing p < 0.05 were included in a multivariable model. Age, sex, education, marital status, living arrangement, smoking status, alcohol consumption, BMI, physical activity, inflammation status, number of chronic conditions, OA (in at least one site between hands, knees and hips), and GDS score were considered as possible independent variables. Based on biological plausibility, we also tested multiplicative interaction terms (OA × sex, and OA × GDS) to explore potential effect modification. Adjusted Odds Ratios (OR) were presented with the corresponding 95% confidence intervals (CI). As sensitivity analysis, we also tested the inclusion of pain (in at least one site between hands, knees, and hips) as a covariate. Model fits were compared using likelihood-ratio tests and Akaike Information Criterion (AIC).

To elucidate whether the relationship between OA and MMSE trajectories over time was direct or mediated by GDS score, structural equation modeling (SEM) was evaluated, with the hypotheses that GDS score was related to MMSE trajectories and that OA was associated with both GDS score and MMSE trajectories (Brown et al. 2002; James & Ferguson 2020). Adequacy of model fit was assessed considering chi-square test, standardized root mean square residual (SRMR) and goodness-of-fit index (GFI).

All analyses were performed using SAS software version 9.4 (SAS Institute Inc., Cary, NC, USA), and a p-value < 0.05 was considered statistically significant.

## Results

The study sample included 2,945 older adults, among whom 1,864 (63.3%) had OA and 1,081 (36.7%) did not (Table 1). Compared to participants without OA, those with OA were older (76.9 ± 7.7 vs. 74.2 ± 7.3 years, *p* < 0.001), and more likely to be female (65.8% vs. 47.7%, *p* < 0.001). Additionally, OA was associated with higher GDS scores (9.7 ± 6.5 vs. 7.3 ± 5.7, *p* < 0.001) and lower MMSE scores at baseline (23.0 ± 5.7 vs. 24.3 ± 5.1, *p* < 0.001).

**Table 1:** Baseline characteristics of the study participants by OA

	All (n = 2945)	OA		*p*-value
		No OA (n = 1081)	OA (n = 1864)	
Age, years, mean ± SD	80.45 (7.93)	75.68 (7.65)	83.22 (6.67)	< 0.001
Sex, females, n (%)	1743 (59.2)	516 (47.7)	1227 (65.8)	< 0.001
Education, ≤ 5 years, n (%)	2585 (87.8)	917 (84.8)	1668 (89.5)	< 0.001
Living arrangements, n (%)				
Living with somebody	2368 (80.7)	908 (84.2)	1460 (78.6)	< 0.001
Living alone	506 (17.2)	163 (15.1)	343 (18.5)	
Living in nursing home	62 (2.1)	8 (0.7)	54 (2.9)	
Smoking habits, n (%)				< 0.0001
Never	1799 (61.1)	566 (52.4)	1233 (66.2)	
Former	877 (29.8)	371 (34.4)	506 (27.2)	
Current	267 (9.1)	143 (13.2)	124 (6.7)	
Alcohol consumption, n (%)				
No or occasional	2054 (69.8)	683 (63.2)	1371 (73.6)	< 0.001
Light to moderate	530 (18.0)	226 (20.9)	304 (16.3)	
Heavy	361 (12.2)	172 (15.9)	189 (10.1)	
BMI, mean ± SD	27.6 ± 4.6	26.9 ± 4.1	28.0 ± 4.8	< 0.001
N. chronic diseases, median (IQR)	3 (2, 5)	3 (2, 4)	4 (2, 5)	< 0.001
N. chronic diseases (excluding OA), median (IQR)	3 (2, 4)	3 (2, 4)	3 (2, 4)	< 0.001
MMSE, mean ± SD	24.3 ± 5.5	24.3 ± 5.1	23.0 ± 5.7	< 0.001
GDS, mean ± SD	8.8 ± 6.3	7.3 ± 5.7	9.7 ± 6.5	< 0.001
ADL > 2, n (%)	529 (19.8)	191 (19.5)	338 (20.0)	0.750
Physical Activity ≥ 4 h/week, n (%)	1246 (57.7)	556 (67.1)	690 (51.8)	< 0.001
High inflammatory status, n (%)	1354 (49.5)	439 (43.3)	915 (53.2)	< 0.001

*IQR* Interquartile Range, *OA* Osteoarthritis, *SD* Standard Deviation, *BMI* Body Mass Index, *MMSE* Mini-Mental State Examination, *GDS* Geriatric Depression Scale, *ADL* Activities of Daily Living

**Fig. 1:**
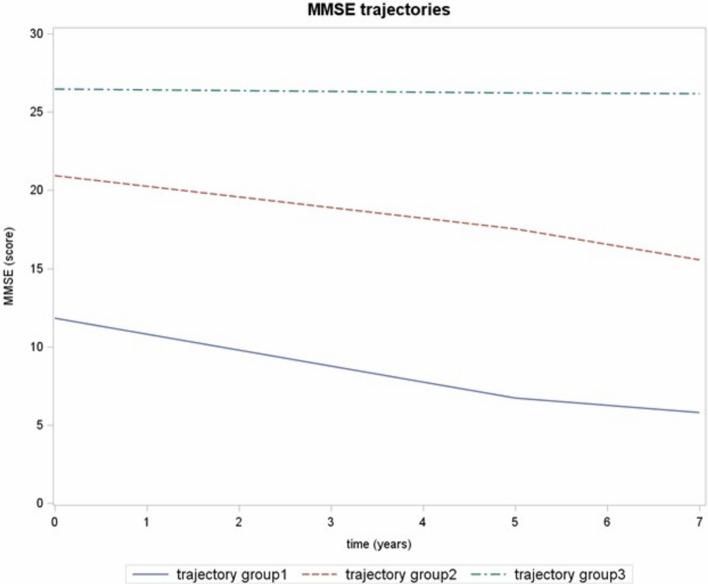
Mini-Mental State Examination (MMSE) trajectories patterns identified. Trajectory group1: severe cognitive decline; Trajectory group2: moderate cognitive decline; Trajectory group3: stability in MMSE score

Over the 7-year follow-up, three distinct cognitive trajectories were identified based on MMSE scores (Fig. 1; Supplementary Table 1 and 2): Trajectory 1 (severe cognitive decline, n = 261; MMSE score at the baseline 11.5 ± 5.7, at the first follow-up 6.2 ± 4.4, at the second follow-up 5.8 ± 3.8), Trajectory 2 (moderate decline, n = 865; MMSE score at the baseline 20.6 ± 3.2, at the first follow-up 17.3 ± 3.9, at the second follow-up 15.2 ± 4.8), and Trajectory 3 (stability, n = 1819; MMSE score at the baseline 26.5 ± 2.5, at the first follow-up 26.2 ± 2.4, at the second follow-up 26.1 ± 2.8). The mean posterior membership probabilities were 0.91 ± 0.13 for the severe decline group, 0.86 ± 0.13 for the moderate decline group, and 0.94 ± 0.11 for the stable group. The proportion of participants with membership probability ≥ 0.7 was 89.3%, 82%, and 93.2%, respectively, indicating good classification quality across groups. All three trajectories were best characterized by linear functions. Model adequacy was supported by fit statistics (BIC), average posterior probabilities (≥ 0.7 for all groups), and relative entropy (0.82), which indicated good separation among the three trajectories. Participants in Trajectory 1 were the oldest (85.1 ± 6.4 years), had the lowest education levels, and exhibited the highest burden of multimorbidity (median 5 chronic diseases, *p* < 0.001, Table 2). OA was more frequent among individuals with greater cognitive decline, with 72.8% of those in Trajectory 1 and 72.6% in Trajectory 2 affected, compared to 57.5% in Trajectory 3 (*p* < 0.001). Multivariable logistic regression analyses (Table 3) showed that OA was significantly associated with an increased likelihood of moderate cognitive decline (Trajectory 2 vs. 3: OR = 1.31, 95% CI: 1.03–1.71, *p* = 0.039), though no significant association was found for severe decline (Trajectory 1 vs. 3: OR = 1.31, 95% CI: 0.71–2.43, *p* = 0.388). Interaction terms OA × sex, and OA × GDS, evaluated to explore potential effect modification, were not significant (*p* = 0.799 and *p* = 0.382, respectively). Given the central role of pain in the theoretical framework linking OA to cognitive decline, we further examined its role in two complementary analyses. First, when pain was added as a covariate in the multivariable logistic regression model (sensitivity analysis, Supplementary Table 3), it was not significantly associated with cognitive trajectories (OR = 1.14, 95% CI 0.86–1.53, *p* = 0.343 for severe cognitive decline vs stability; OR = 0.96, 95% CI 0.48–1.91, *p* = 0.903 for moderate decline vs stability). Model fit did not improve with the inclusion of pain (likelihood-ratio test *p* = 0.311; AIC increased by 3), and the effect of OA was attenuated (OR = 1.29, 95% CI 0.99–1.68, *p* = 0.055), suggesting only a marginal influence of pain on the association.

**Table 2:** Baseline characteristics of the study participants according to MMSE trajectories identified

	Trajectory 1 Severe cognitive decline (n = 261)	Trajectory 2 Moderate cognitive decline (n = 865)	Trajectory 3 Stability (n = 1819)	*p*-value
Age, years, mean ± SD	85.1 ± 6.4	80.6 ± 6.7	72.4 ± 5.7	< 0.001
Sex, females, n (%)	175 (67.1)	556 (64.3)	1012 (55.6)	< 0.001
Education, ≤ 5 years, n (%)	248 (95.4)	831 (96.1)	1506 (82.8)	< 0.001
Living arrangements, n (%)				< 0.001
Living with somebody	208 (80.6)	660 (76.7)	1500 (82.6)	
Living alone	25 (9.7)	177 (20.5)	304 (16.7)	
Living in nursing home	25 (9.7)	24 (2.8)	13 (0.7)	
Smoking habits, n (%)				< 0.001
Never	203 (78.4)	581 (67.1)	1015 (55.8)	
Former	50 (19.3)	223 (25.8)	604 (33.2)	
Current	6 (2.3)	61 (7.1)	200 (11.0)	
Alcohol consumption, n (%)				
No or occasional	226 (86.6)	632 (73.1)	1196 (65.8)	< 0.001
Light to moderate	25 (9.6)	157 (18.1)	348 (19.1)	
Heavy	10 (3.8)	76 (8.8)	275 (15.1)	
BMI, mean ± SD	26.6 ± 4.9	27.4 ± 4.7	27.8 ± 4.5	< 0.001
Hand OA, n (%)	104 (39.9)	412 (47.6)	650 (35.7)	< 0.001
Knee OA, n (%)	148 (56.7)	468 (54.1)	800 (38.5)	< 0.001
Hip OA, n (%)	125 (47.9)	316 (36.5)	435 (23.9)	< 0.001
OA, n (%)	190 (72.8)	628 (72.6)	1046 (57.5)	< 0.001
N. chronic diseases, median (IQR)	5 (4, 6)	4 (3, 5)	3 (2, 4)	< 0.001
N. chronic diseases (excluding OA), median (IQR)	4 (3, 5)	3 (2, 4)	2 (1, 4)	< 0.001
MMSE, mean ± SD	11.5 ± 5.7	20.6 ± 3.2	26.5 ± 2.5	< 0.001
GDS, mean ± SD	11.2 ± 6.0	10.8 ± 6.6	7.7 ± 5.9	< 0.001
ADL > 2, n (%)	46 (19.4)	153 (19.6)	330 (20.0)	0.958
Physical Activity ≥ 4 h/week, n (%)	10 (9.5)	187 (32.9)	1058 (71.1)	< 0.001
High inflammatory status, n (%)	170 (70.3)	445 (55.7)	739 (43.7)	< 0.001

*IQR* Interquartile Range, *OA* Osteoarthritis, *SD* Standard Deviation, *BMI* Body Mass Index, *MMSE* Mini-Mental State Examination, *GDS* Geriatric Depression Scale, *ADL* Activities of Daily Living

**Table 3:** Association between Osteoarthritis (OA) and Mini-Mental State Examination trajectories

	Trajectory 1 vs 3 Severe cognitive decline vs stability			Trajectory 2 vs 3 Moderate cognitive decline vs stability		
	OR	95% CI	*p*-value	OR	95% CI	*p*-value
Model 1	1.12	0.79–1.57	0.527	1.32	1.07–1.63	0.009
Model 2	1.31	0.71–2.43	0.388	1.31	1.03–1.71	0.039

*CI* Confidence Interval, *OR* Odds Ratio. Model 1: adjusted for sex and age; Model 2: adjusted for sex, age, education, living arrangements, Body Mass Index-BMI classes, smoking status, drinking habits, number of chronic diseases (excluding OA), Geriatric Depression Scale-GDS score, physical activity, inflammatory status

Second, we evaluated an additional structural equation model to assess whether pain might mediate the OA–cognition relationship. Pain was not significantly associated with MMSE trajectories (β = –0.022, *p* = 0.278), and the indirect effect of OA through pain was not significant (*β* = 0.030, *p* = 0.236), providing no evidence of mediation.

**Fig. 2:**
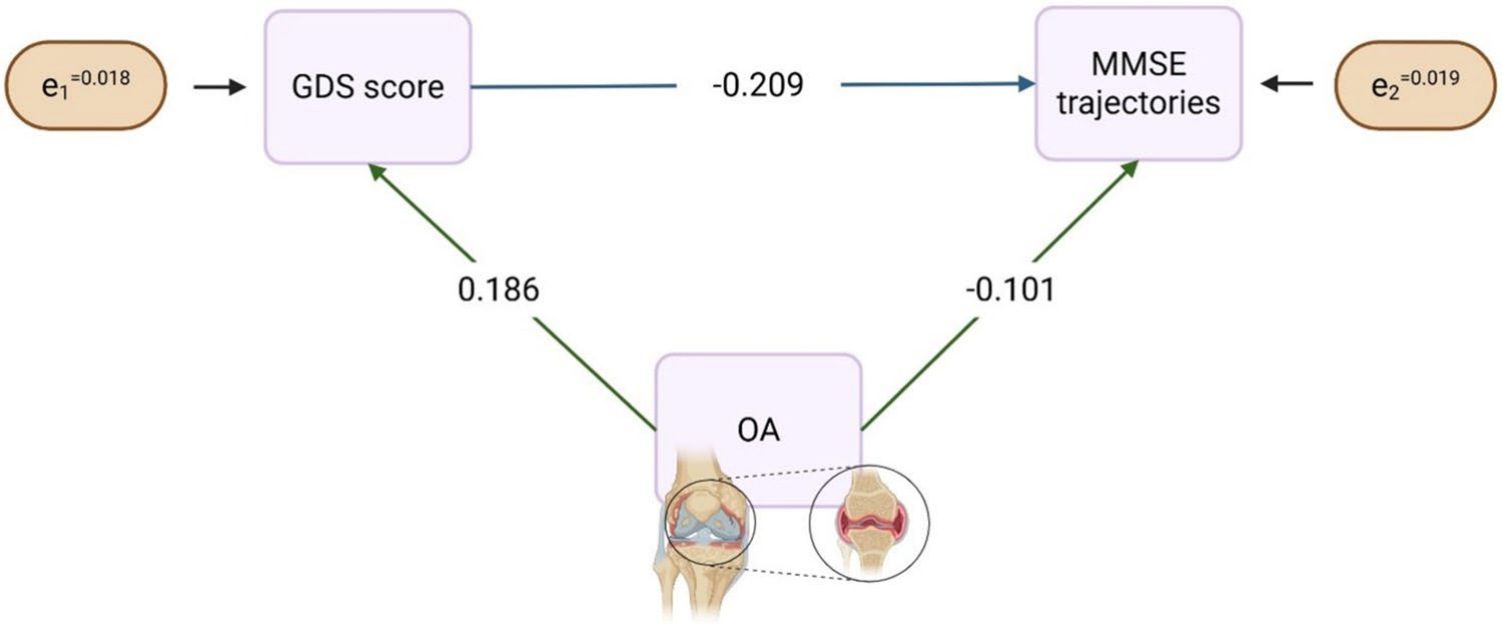
Final structural equation model

Considering an a priori hypothesis model defining GDS score at the baseline and MMSE trajectories as endogenous variables, and OA as exogenous variable, we found that OA was associated with GDS score (beta 0.186, *p* < 0.001) and with MMSE trajectories (beta − 0.101, *p* < 0.001), and that GDS score was also associated with MMSE trajectories (beta − 0.209, *p* < 0.001) (Fig. 2). This suggests that OA is associated with MMSE trajectories both directly and indirectly through GDS score. The direct effect of OA on MMSE trajectories was −0.102, the indirect effect through GDS score was − 0.039, suggesting that about 30% of the total effect of OA on MMSE trajectories was mediated by depression. Both direct and indirect effects were significant. The model provided an excellent fit to the observed data (standardized root mean square residual (SRMR) = 0.000 and goodness-of-fit index (GFI) = 1.000).

## Discussion

This is the first longitudinal study, to our knowledge, to examine MMSE trajectories in relation to both OA and depressive symptoms (measured using GDS) in a large cohort of community-dwelling older adults. We found a higher prevalence of OA among individuals experiencing cognitive decline, with 73% of participants in the moderate and severe decline trajectories compared to 57.5% in the cognitively stable group. OA was significantly associated with increased risk of moderate cognitive decline, both directly and indirectly via depressive symptoms, which accounted for approximately 30% of OA’s total effect.

Our results align with prior studies (Kang et al. 2023; Kazim et al. 2023; Mayburd & Baranova 2019; Wang & Ni 2022) and highlight the complex interaction between OA and mental health, emphasizing the value of multidisciplinary strategies, such as integrated pain management, psychological support, and cognitive stimulation, to help prevent or delay cognitive deterioration.

The association between OA and cognitive decline emerged for the moderate trajectory (Trajectory 2), but not for the most severe decline (Trajectory 1), despite a similar OA prevalence across groups. This pattern is consistent with stage-specific mechanisms: in advanced impairment, decline may be driven predominantly by neurodegenerative processes and overall comorbidity burden, making any additional OA-related effect less detectable. By contrast, OA may contribute more in earlier or intermediate stages. Notably, these estimates were obtained from models adjusted for age, education, multimorbidity, and other covariates.

It is well known that inflammation plays a crucial role in the pathogenic mechanisms of cognitive deterioration and neurodegeneration, functioning as both a causal and consequent process (Myasoedova et al. 2024). Although systemic inflammation is less pronounced in OA compared to other rheumatic diseases, it remains an inflammatory chronic condition. Neuroinflammation can be triggered by neuronal death, but at the same time, it can further fuel the progression of neurodegeneration. Neuropathological studies have shown that the inflammatory response in the central nervous system is an early event in Alzheimer’s disease, acting synergistically with β-amyloid deposition, tau pathology, and neuronal loss (Stakos et al. 2020). At the systemic level, another relevant mechanism is inflammaging —a state of low-grade chronic inflammation increasingly recognized as a risk factor for cognitive decline (Ferrucci & Fabbri 2018). This condition involves elevated levels of inflammatory markers such as C-reactive protein (CRP) and interleukin-6 (IL-6), along with immune system dysregulation. Preclinical studies suggest that inflammaging increases blood–brain barrier permeability, activates microglia, and promotes the release of pro-inflammatory cytokines (e.g., IL-1, IL-6, TNF), triggering neurodegenerative pathways (Fard et al. 2022). Consistent evidence from both cross-sectional and longitudinal studies links high systemic inflammation with poorer cognitive performance across multiple domains, including memory, executive function, language, and visuospatial skills (Fard et al. 2022; Myasoedova et al. 2024).

Another critical factor in the relationship between OA and cognitive decline is chronic pain, impacting not only physical but also emotional and cognitive domains. It is frequently associated with depression, social isolation, and reduced independence, contributing to increased cognitive vulnerability, particularly in older adults (Chen et al. 2023). Neuroimaging studies have identified structural and functional changes in brain regions like the hippocampus and prefrontal cortex—key areas for memory and executive function. For instance, patients with knee OA have shown hippocampal volume loss and increased activity despite being cognitively normal at the time of MRI, suggesting early neural alterations linked to dementia risk (Liu et al. 2023). Several hypotheses explain the pain–cognition link. One widely supported theory is that chronic pain competes for cognitive resources, diverting attention and memory capacity and impairing functions such as executive control and focus (Landrø et al. 2013; Samartin-Veiga et al. 2019). Additionally, pain-related reductions in mobility and activity levels may lead to social withdrawal and decreased brain stimulation, further elevating cognitive risk by limiting neuroplasticity (Cai et al. 2023). A further consideration is the potential cognitive impact of medications used to treat chronic pain. Although not directly assessed in our analysis, long-term use of opioids and sedatives has been associated with cognitive impairment, especially in older adults (Warner et al. 2023). While acute use may affect short-term cognition, concerns persist about their lasting effects, though disentangling drug effects from those of pain or OA itself remains difficult and evidence remains inconclusive (Warner et al. 2023). Our sensitivity analysis considering also pain, we found that it was not independently associated with cognitive decline, and including pain did not improve model fit, but it attenuated the association between OA and severe cognitive decline.

Our findings indicate that OA is associated with MMSE trajectories both directly and indirectly through the GDS score, suggesting that approximately 30% of the total effect of OA on cognitive decline is mediated by depressive symptoms. The remaining 70% of the effect appears to be direct and independent of depression. These results contribute to the growing body of research on the interplay between physical and mental health factors in cognitive aging, adding an important piece to the chain of mechanisms potentially linking OA and cognitive decline. While the association between depression and cognitive decline is well-documented, evidence regarding OA's impact remains more limited (Guo et al. 2022). Some studies have found no significant or direct relationship, suggesting that depression might be the primary driver of cognitive outcomes (Zhao et al. 2025). Depression, particularly during acute phases, is linked to deficits in memory, executive function, attention, and psychomotor speed. Neurobiological alterations, such as hypothalamic–pituitary–adrenal (HPA) axis dysregulation and elevated cortisol levels, contribute to neurotoxicity and hippocampal dysfunction (Høifødt et al. 2019). Additionally, reduced levels of brain-derived neurotrophic factor (BDNF) are frequently associated with depressive symptoms and play a critical role in cognitive and mood regulation (Correia et al. 2023).

### Limitations and strengths

Our study has several limitations that should be acknowledged. First, its observational design restricts the ability to establish causal relationships between OA, depressive symptoms, and cognitive decline. Although the cohort is large and diverse, it is drawn from two provinces in northern Italy, which may limit the generalizability of the findings to other populations. Additionally, we lacked data on specific pharmacological treatments for depression, which may influence cognitive outcomes and could not be accounted for in the analyses. The MMSE, although widely used, does not provide a comprehensive assessment of cognitive function and may overlook key executive domains. Reliance on self-reported information for certain variables (e.g., physical activity, alcohol consumption) introduces the possibility of recall bias. Moreover, individuals with incomplete baseline data were excluded, potentially introducing selection bias. Importantly, the possibility of joint-specific or disease-burden–specific associations warrants consideration. While we performed exploratory analyses stratified by joint site (hand, hip, knee) and by the number of affected joints, these analyses were limited by insufficient statistical power and therefore were not presented in detail. This limitation underscores the need for future studies with larger samples to better evaluate potential heterogeneity in the relationship between OA patterns and cognitive trajectories. Another limitation concerns the number of MMSE assessments available. Group-based trajectory modeling allows inclusion of participants with only a single observation under the missing-at-random assumption. We retained individuals with only baseline MMSE data to preserve the representativeness of the cohort and avoid selection bias. However, these individuals contribute limited information on within-person change, which may slightly reduce the precision of estimated trajectory shapes. Future research with more complete longitudinal follow-up is warranted to validate the trajectory patterns observed. A further methodological consideration is the use of the conventional “classify–analyze” approach, whereby participants were assigned to their most likely trajectory group without posterior probability weighting in subsequent models. Given the high average posterior probabilities and entropy values, the risk of substantial misclassification bias is likely limited.

Despite these limitations, the study benefits from a longitudinal design with 7 years of follow-up, allowing us to characterize cognitive trajectories over time. The large sample size and stratified random sampling enhance both representativeness and statistical power, supporting the robustness of the findings.

## Conclusions

In conclusion, this study highlights the significant association between OA, depression, and cognitive decline in older adults. Our findings suggest that depression partially mediates the impact of OA on cognitive trajectories, underscoring the importance of addressing mental health in the management of OA. However, further research is needed to explore the underlying mechanisms and confirm causal pathways.

## Supplementary Information

Below is the link to the electronic supplementary material.

Supplementary file 1

## Data Availability

No datasets were generated or analysed during the current study.
