# Supplementary material for: The impact of osteoarthritis and geriatric depression scale on mini-mental state examination trajectories over seven years
Source: Eur J Ageing. 2025 Dec 16;23(1):4. doi: 10.1007/s10433-025-00900-x (PMC12775186; doi:10.1007/s10433-025-00900-x)
Supplement: Supplementary file 1 [file 10433_2025_900_MOESM1_ESM.docx]

**Supplementary Table 1.** Trajectory modeling results

| **Model** | **BIC** | **2ΔBIC** | **groups with**  **membership probabilities**  **of at least 5%** | **within each group average**  **posterior probability**  **of membership ≥ 0.7** |
| --- | --- | --- | --- | --- |
| 1-group | -17820.90 | - | Yes | Yes |
| 2-group | -16876.37 | 1889.06 | Yes  (24.0%; 76.0%) | Yes  (0.89; 0.96) |
| 3-group | -16523.46 | 705.82 | Yes  (8.1%; 26.4%; 65.4%) | Yes  (0.88; 0.85; 0.94) |
| 4-group | -16274.10 | 498.72 | No  (3.4%; 14%; 25%; 57.6%) | No  (0.95; 0.83; 0.69; 0.93) |
| 5-group | -16290.22 | 466.48 | No  (3.3%, 13.9%; 25%; 57%; 0.8%) | No  (0.94; 0.83; 0.68; 0.92; 0.51) |

*Abbreviations:* Bayesian Information Criterion (BIC)

Based on model fit indices, the 3-trajectory model was selected as the optimal solution. Although models with four and five trajectories showed slightly better BIC values, they failed to meet the criteria on group membership , as one or more groups had less than 5% of participants and average posterior membership probabilities below 0.7. The 3-group model satisfied all recommended criteria, including BIC improvement, minimum group size, and acceptable classification quality, and was therefore retained as the final model.

**Supplementary Table 2.** Final model characteristics

| **Trajectory** | **Shape** | **n (%)** | **Mean posterior**  **probability±SD** | **% participants with membership probability ≥0.7** | **Baseline MMSE**  **mean±SD** | **Intercept** | **Linear term** |
| --- | --- | --- | --- | --- | --- | --- | --- |
| Group 1, Severe cognitive decline | Linear | 261 (8.9) | 0.91±0.13 | 233/261 (89.3) | 11.5±5.7 | 11.4 (p<0.001) | -1.04 (p<0.001) |
| Group 2, Moderate cognitive decline | Linear | 865 (29.4) | 0.86±0.14 | 709/865 (82.0) | 20.6±3.2 | 20.3 (p<0.001) | -0.66 (p<0.001) |
| Group 3, Stability | Linear | 1819 (61.7) | 0.94±0.11 | 1695/1819 (93.2) | 26.2±2.4 | 26.3 (p<0.001) | -0.04 (p=0.036) |

**Supplementary Table 3.** Association between Osteoarthritis (OA) and Mini-Mental State Examination trajectories, sensitivity analysis including also pain as covariate

|  | **Trajectory 1 vs 3**  **Severe cognitive decline**  **vs stability** | | | **Trajectory 2 vs 3**  **Moderate cognitive decline**  **vs stability** | | |
| --- | --- | --- | --- | --- | --- | --- |
|  | **OR** | **95% CI** | **p-value** | **OR** | **95% CI** | **p-value** |
| Model 1 | 1.14 | 0.86-1.53 | 0.343 | 0.96 | 0.48-1.91 | 0.903 |
| Model 2 | 1.18 | 0.57-2.43 | 0.657 | 1.29 | 0.99-1.68 | 0.055 |

*Abbreviations*: CI, Confidence Interval; OR, Odds Ratio

Model 1: adjusted for sex and age; Model 2: adjusted for sex, age, education, living arrangements, Body Mass Index-BMI classes, smoking status, drinking habits, number of chronic diseases (excluding OA), Geriatric Depression Scale-GDS score, physical activity, inflammatory status, pain (hands, knee, hip).
